# A robust multi-objective optimization framework to capture both cellular and intercellular properties in cardiac cellular model tuning: Analyzing different regions of membrane resistance profile in parameter fitting

**DOI:** 10.1371/journal.pone.0225245

**Published:** 2019-11-15

**Authors:** Elnaz Pouranbarani, Rodrigo Weber dos Santos, Anders Nygren

**Affiliations:** 1 Department of Electrical and Computer Engineering, University of Calgary, Calgary, Alberta, Canada; 2 Department of Computer Science and the Graduate Program of Computational Modeling, Federal University of Juiz de Fora, Juiz de Fora, Minas Gerais, Brazil; University of New South Wales, AUSTRALIA

## Abstract

Mathematical models of cardiac cells have been established to broaden understanding of cardiac function. In the process of developing electrophysiological models for cardiac myocytes, precise parameter tuning is a crucial step. The membrane resistance (*R*_*m*_) is an essential feature obtained from cardiac myocytes. This feature reflects intercellular coupling and affects important phenomena, such as conduction velocity, and early after-depolarizations, but it is often overlooked during the phase of parameter fitting. Thus, the traditional parameter fitting that only includes action potential (AP) waveform may yield incorrect values for *R*_*m*_. In this paper, a novel multi-objective parameter fitting formulation is proposed and tested that includes different regions of the *R*_*m*_ profile as additional objective functions for optimization. As *R*_*m*_ depends on the transmembrane voltage (*V*_*m*_) and exhibits singularities for some specific values of *V*_*m*_, analyses are conducted to carefully select the regions of interest for the proper characterization of *R*_*m*_. Non-dominated sorting genetic algorithm II is utilized to solve the proposed multi-objective optimization problem. To verify the efficacy of the proposed problem formulation, case studies and comparisons are carried out using multiple models of human cardiac ventricular cells. Results demonstrate *R*_*m*_ is correctly reproduced by the tuned cell models after considering the curve of *R*_*m*_ obtained from the late phase of repolarization and *R*_*m*_ value calculated in the rest phase as additional objectives. However, relative deterioration of the AP fit is observed, demonstrating trade-off among the objectives. This framework can be useful for a wide range of applications, including the parameters fitting phase of the cardiac cell model development and investigation of normal and pathological scenarios in which reproducing both cellular and intercellular properties are of great importance.

## Introduction

Mathematical models of cardiac electrophysiology are of the utmost importance in solving a wide range of biomedical and pharmacological problems [1]. Computational models of cardiac myocytes facilitate the understanding of important properties and phenomena at both cellular and tissue levels [2]. Cardiac myocyte models are usually represented by non-linear systems of ordinary differential equations (ODEs), in which a large number of unknown parameters need to be tuned during the processes of model development and calibration [3, 4].

For tuning the parameters, several optimization methods have been proposed to overcome the high computational burden and inaccuracy of a trial and error approach [2]. Gradient and heuristic-based approaches [5–8] are the most common methods for tuning the model parameters. In accordance with the literature review, one can notice that the optimization approaches developed for tuning cardiac cell models are mainly limited to the single objective problems of fitting action potential (AP) waveforms, ionic channel currents, or a combination thereof, i.e., properties of single cardiac cells. These optimization protocols can be inaccurate in reproducing interconnected cell properties, i.e., tissue behavior [3]. However, intercellular electrical coupling properties are critical in the context of large-scale modeling [9].

To illustrate the importance of considering intercellular features, it has been demonstrated that two distinct sets of parameters can generate identical single-cell AP waveforms [10, 11], while their intercellular characteristics are different [3]. This dissimilarity stems from the incomplete characterization of current dependencies in source-sink relationships [3]. AP propagation in the tissue occurs provided that an adequate charge is delivered to local sinks by active sources [12]. Membrane resistance (*R*_*m*_) is a useful indicator to characterize this intercellular communication [13, 14].

Weidmann [15] pioneered the measurement of *R*_*m*_ during AP waveform and described a phenomenon called all-or-nothing repolarization (ANOR) in sheep Purkinje fiber. To demonstrate all-or-none behavior, current pulses were applied after the absolute refractory period to perturb the AP trajectory. In response to these pulses, the AP either returned to the diastolic phase (repolarized) or exhibited regenerative depolarization to follow the AP waveform. This behavior, in which a perturbation of membrane voltage during the AP leads to repolarization, is known as auto-regenerative ANOR [16, 17].

To further investigate important dynamic characteristics, including *R*_*m*_ and auto-regenerative ANOR, a three-dimensional (3D) visualization of time, transmembrane voltage, and current (*t-V*_*m*_*-I*_*m*_) was introduced by Zaniboni [17–19]. In these studies, it has been demonstrated that negative *R*_*m*_ [20] occurs in the late phase of repolarization. The time interval during which *R*_*m*_ becomes negative is called the ANOR window, and its importance in the occurrence of early after-depolarizations (EADs) has been clearly shown [17]. The membrane during this time interval behaves as a source of current, which results in surges in outward currents in response to any additional hyperpolarizing current. Similarly, depolarizing perturbations in voltage result in additional depolarizing current, which may cause EADs. Moreover, on the borders of the ANOR window, high values of *R*_*m*_ and nonlinearity of the *I*_*m*_*-V*_*m*_ curve appear. In the *R*_*m*_ profiles of similar APs obtained with different sets of parameters in the same model or *R*_*m*_ profiles of different models with similar cell types, it has been demonstrated that these high *R*_*m*_ values can occur at different voltages [17]. Furthermore, it has been shown that measuring a high value of *R*_*m*_ at the end of plateau is computationally unfeasible and can be affected by discontinuities of the *R*_*m*_ profile [21]. However, these observations have not been directly considered in the cellular fitting approach described in this paper.

The speed of AP waveform propagation is called conduction velocity (*θ*). Regions of cardiac tissue with slower *θ* are at risk of re-entrant propagation, which is associated with arrhythmia [22]. Also, the relationship between *R*_*m*_ during the diastolic phase (i.e., rest phase) and *θ*, in a human atrial model was studied in [23], which has been demonstrated that decreasing *R*_*m*_ results in reduced *θ*. This reduction results from the increase in the electrical load on neighboring cells. Moreover, the analysis of a human ventricular model suggests that reduction of [K^+^]_o_ results in increased *R*_*m*_ during the rest phase, which can be a symptom of hypokalemia [24].

The discussion above emphasizes the benefits and rationale for including *R*_*m*_ as one of the objective functions during parameter fitting. In [3], Kaur et al. considered *R*_*m*_ values together with AP as objectives for multi-objective optimization. However, Kaur et al. [3] did not account for the fact that *R*_*m*_ values as a function of the transmembrane voltage, *V*_*m*_, increase steeply to become singular at specific values of *V*_*m*_. The dependency of the *R*_*m*_ profile on *V*_*m*_ and discontinuity of this profile pose a computational challenge for the fitting procedure that must be addressed to ensure a robust procedure.

In this paper, we propose a careful selection of regions of interest for *R*_*m*_ during the AP waveform. These regions are selected after the analysis of different cardiac myocyte models for the human left ventricle under different physiological and pathophysiological conditions. During the different phases of the AP, three regions are identified by excluding ranges near the points which are susceptible to singularity, i.e., when *R*_*m*_ goes to infinity. By specifically considering *R*_*m*_ in these regions, the parameter fitting process is more robust. Considering the AP, *R*_*m*_ waveform in the repolarization phase, and *R*_*m*_ value in the rest phase as objectives allows us to explore the trade-offs of objectives among cellular and intercellular properties within the framework of multi-objective optimization. Moreover, to reduce the number of optimization objectives, an interpolation approach is adapted to fit a curve to *R*_*m*_ points, and fitness of this curve instead of individual *R*_*m*_ points is used as one of the objective functions. This enhances the computational tractability of optimization and facilitates visualization of the Pareto front.

## Materials and methods

### Mathematical models and simulation specifications

Two mathematical models of the human ventricular epicardial cell are used in this study for the optimization: the Ten Tusscher et al. (TNNP) model [25], and the Iyer-Mazhari-Winslow (IMW) model [26]. All simulations are carried out in Matlab R2018a [27], using codes of the models available at www.cellml.org. The detailed description of ODEs associated with these human ventricular cell models can be found in [25, 26]. The built-in ode15s solver in Matlab for stiff systems of ODEs is utilized. Also, the *Parallel Computing Toolbox* is used in the simulations to reduce the computational time.

The AP is produced by injecting a 2 ms current pulse with 20 pA/pF amplitude [10]. To reach the steady-state condition, each model is allowed to run for 20 beats at a 1 s interval (1 Hz), and the 20^th^ AP is selected for analysis. Values of *R*_*m*_ are normalized to a cell capacitance of 180 pF [17, 18].

The TNNP and different configurations of IMW models are considered as the *base* and *reference* (experimental data) *models*, respectively. In other words, key parameters of the *base model* (TNNP) are adjusted to attempt to reproduce data generated by the *reference model* (IMW). The importance of selected parameters is thoroughly discussed in [10]. In addition to the mentioned models, another state-of-the-art ventricular model [28] is utilized to further examine the *R*_*m*_ profile for the proper selection of regions of *R*_*m*_ far from singular points.

### *R*_*m*_ measurement

**Fig 1:**
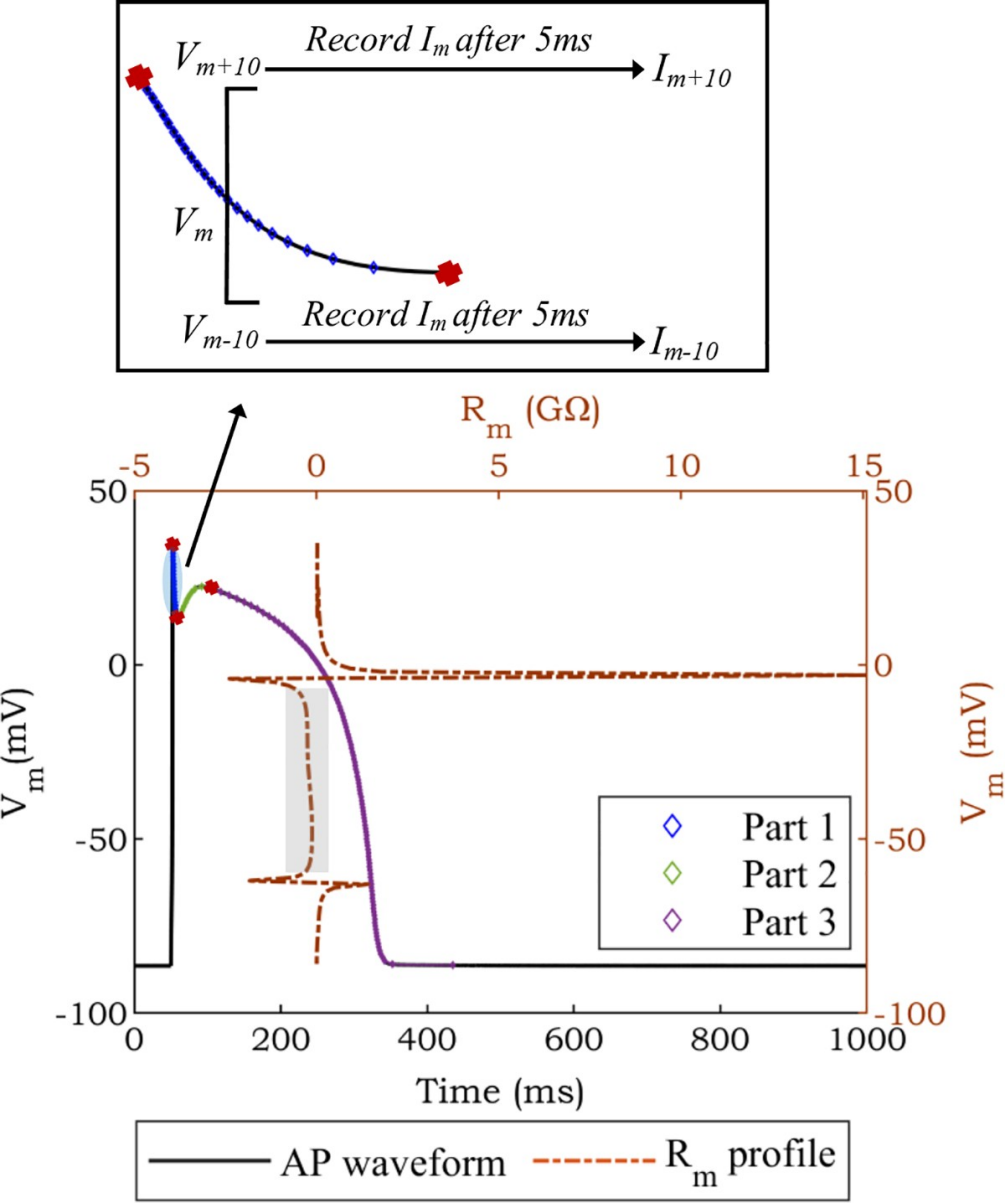
Calculation of *R*_*m*_ during the AP waveform of the TNNP model. The inset illustrates the voltage clamp for a specific point of voltage (*V*_*m*_). According to the existence of local/global minima and maxima (red marks), the AP waveform is separated into three parts. The grey rectangle represents the ANOR region.

The protocol used to measure *R*_*m*_ during the AP waveform at a particular point (voltage) during repolarization is illustrated in Fig 1. For each specific *V*_*m*_ value of interest, the ODEs of the model are solved in two separate simulations. Both simulations start by simulating an AP until reaching the point (voltage) of interest during repolarization. At this point, the simulation is changed to voltage-clamp mode, and the voltage is kept constant at 10 mV higher (*V*_*m+10*_) or lower (*V*_*m-10*_) than the point of interest, respectively. The resulting membrane currents (*I*_*m+10*_ and *I*_*m-10*_) are recorded after 5 ms of voltage-clamp in each simulation. The ratio of difference in voltage (Δ*V*_*m*_) to change in current (Δ*I*_*m*_) is used to estimate *R*_*m*_ [3, 14].

To avoid ambiguities where the same voltage value occurs more than once during repolarization, the AP waveform is divided into three parts based on global/local minima and maxima (red marks in Fig 1). In each part, the voltage clamp protocol described above is applied. Fig 1 represents the *R*_*m*_ profile (brown line) calculated from the AP waveform of the TNNP model as an example. In the *R*_*m*_ profile, the ANOR window (grey rectangle) [17] is bounded by the sharp increase of positive and negative *R*_*m*_ values. Inside the window, *R*_*m*_ values are negative.

It is worth noting that the *R*_*m*_ profile is constructed as a function of voltage, rather than specific points in time. This allows a more direct comparison between models even when there may be a substantial discrepancy between models in the timing of different states of the AP (i.e., substantial differences in AP shape and duration) [3].

### Discontinuity and voltage dependence of the *R*_*m*_ profile

**Fig 2:**
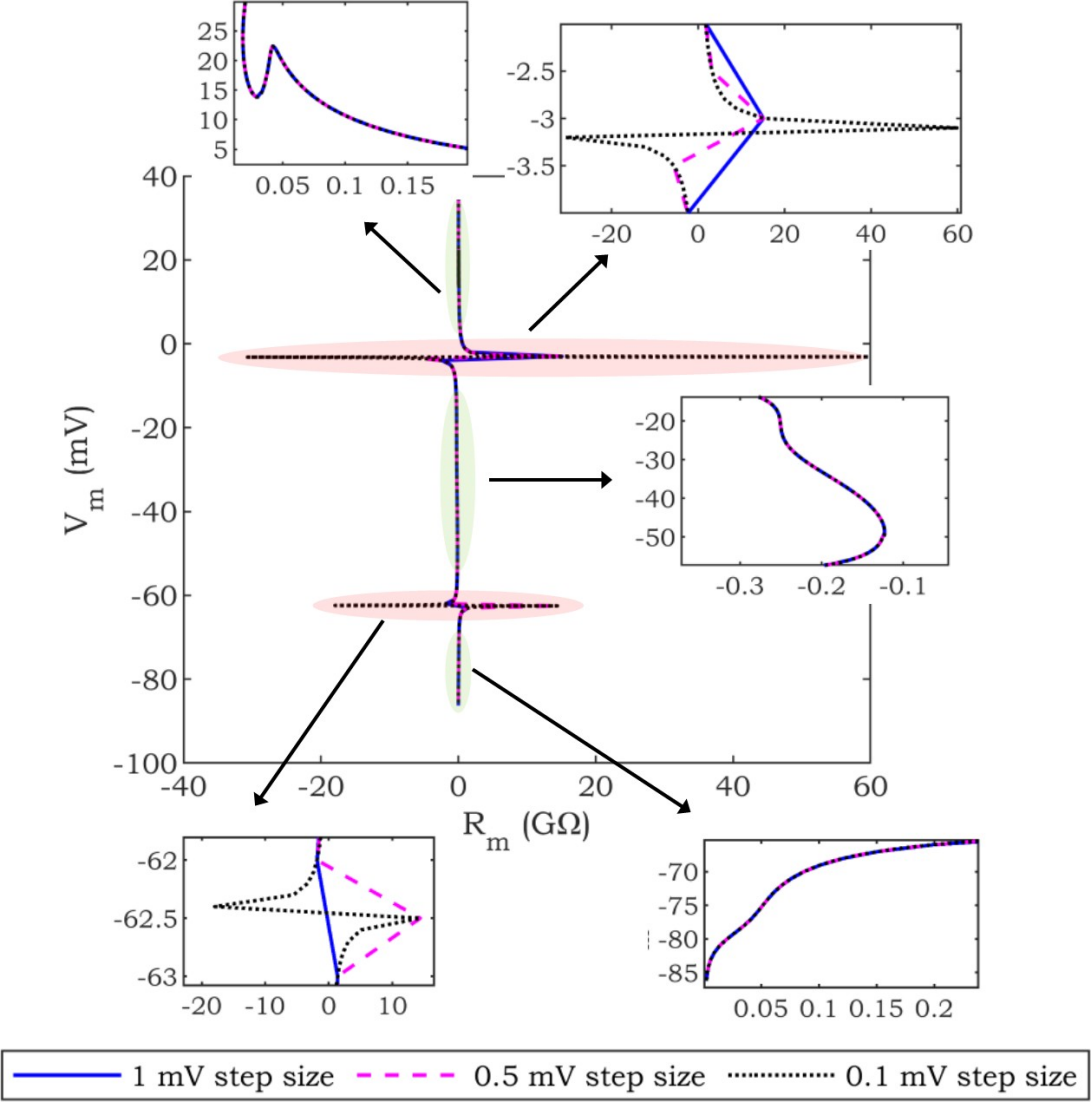
An illustration of discontinuity. The discontinuity of the *R*_*m*_ profile of the TNNP model is shown by voltage clamp in different step sizes (1 mV, 0.5 mV, 0.1 mV). Red and green ovals demonstrate the areas with and without discontinuity, respectively.

As illustrated in Fig 1, *R*_*m*_ is positive during the plateau phase of the AP, becomes negative during the ANOR window, and returns to positive values during late repolarization. The transition from positive to negative values (and the reverse) is associated with a discontinuity due to singular *R*_*m*_ points, i.e., the magnitude of *R*_*m*_ increases, rapidly approaching infinity. Fig 2 provides further insight into the rapidly changing *R*_*m*_ near this discontinuity. In this figure, the *R*_*m*_ profile of the TNNP model is calculated by applying voltage clamp using a range of sampling resolutions (1 mV, 0.5 mV, 0.1 mV). Fig 2 confirms that decreasing the step size to record high resolution of *R*_*m*_ profiles results in increasing peak values for *R*_*m*_ (red oval). Therefore, depending on the *R*_*m*_ step size, different peak values of *R*_*m*_ will be calculated (approaching infinity as the voltage step size decreases). Hence, attempts to match the *R*_*m*_ profile for parameter tuning near these transition points cannot be expected to yield robust results. However, as can be perceived from the parts of *R*_*m*_ profile highlighted by green ovals in Fig 2, this is not a concern other than in the immediate vicinity of the discontinuities.

**Fig 3:**
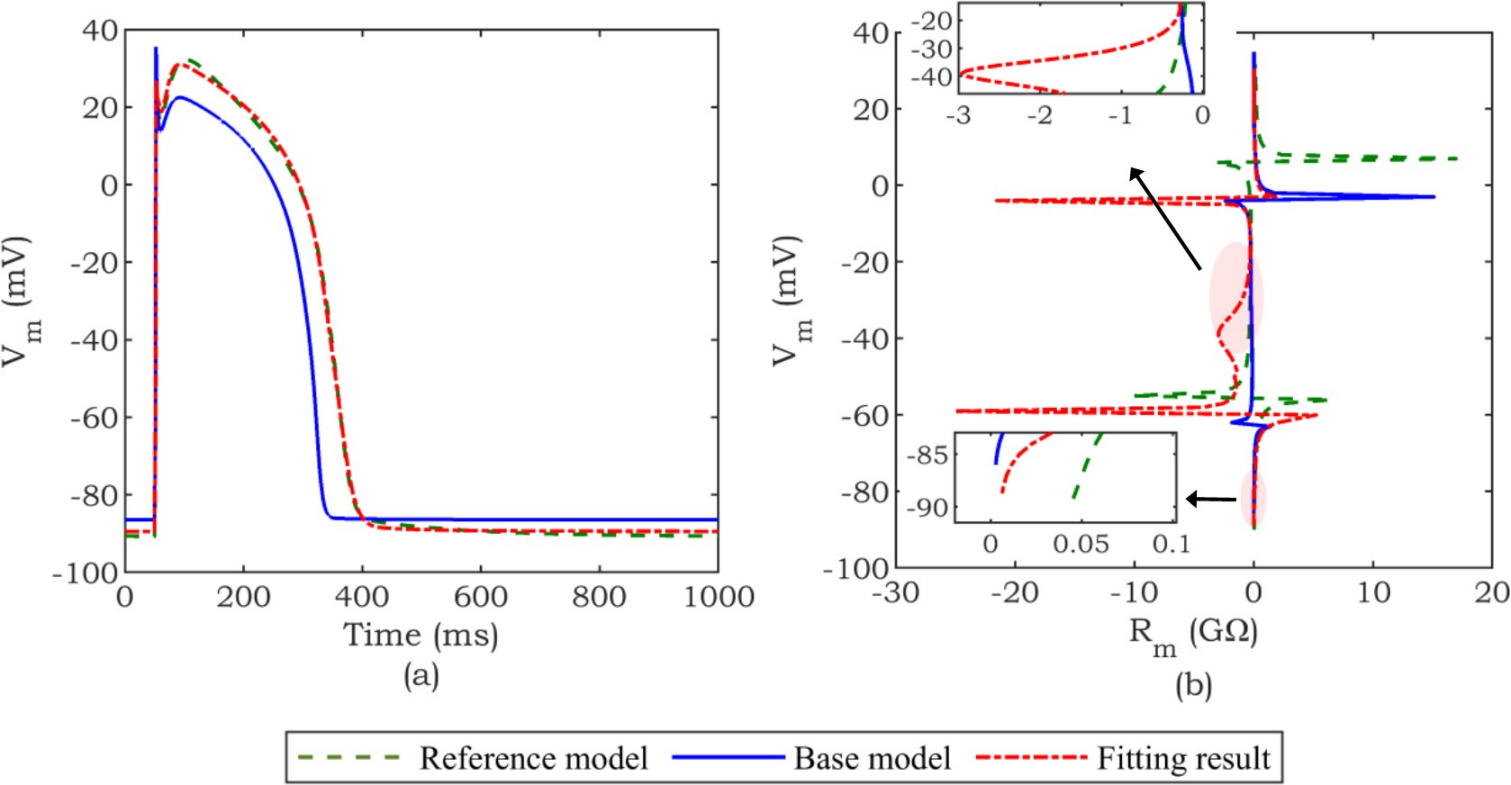
Representation of similar AP with dissimilar *R*_*m*_ profile. (a) AP waveform: The *base model* (TNNP) is fitted to the *reference model* (IMW). (b) *R*_*m*_ profile: *R*_*m*_ profile associated with the fitted *base model* (fitting result) is significantly different from the *R*_*m*_ profile corresponding to the *reference model*.

The challenge described above is further compounded by the fact that *R*_*m*_ points, which are susceptible to be singular, appear at different values of *V*_*m*_ for different cell models, even when the cell models have similar AP (Fig 3(A)). This can be observed in Fig 3(B), which shows that the peak values of the *R*_*m*_ profile of the fitted *base mode*l (fitting result) are not aligned with the peak values of the *reference model* (IMW).

Based on the above discussion, it is important to carefully select the best regions of *R*_*m*_ to use for parameter fitting. This is performed by examining different cell models in different states, as described next.

### Defining allowed and disallowed regions for capturing *R*_*m*_ in optimization

**Fig 4:**
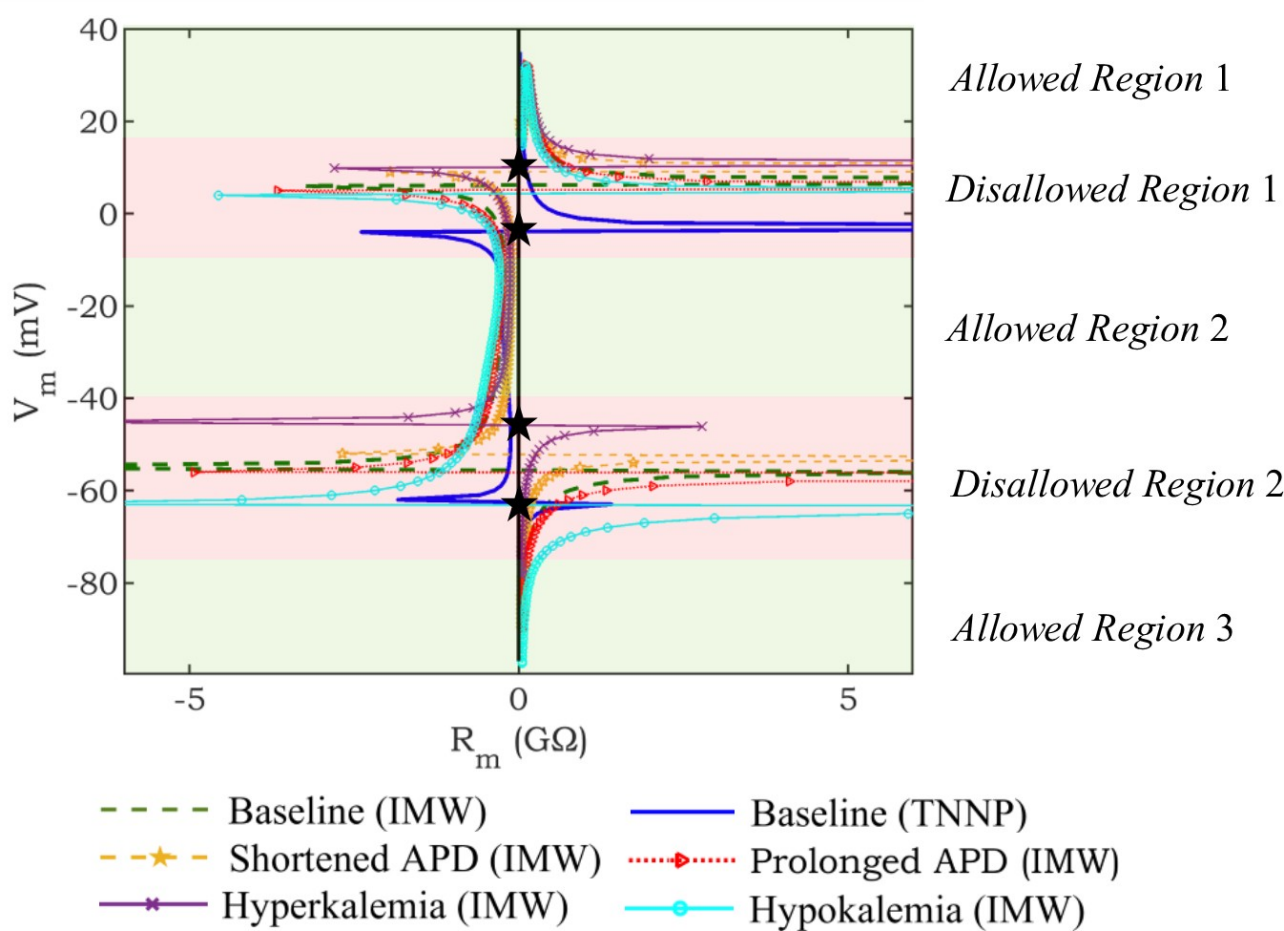
Defining allowed and disallowed regions. *R*_*m*_ profiles of human ventricular cell models (IMW, TNNP, Shortened APD (IMW), Prolonged APD (IMW), Hyperkalemia (IMW), and Hypokalemia (IMW) models) are demonstrated to define allowed (green regions) and disallowed (red regions) regions. Disallowed regions are defined based on the location of black pentagrams where singularities occur.

To establish specific disallowed regions for our models of interest, for each model configuration of interest, we first find the singular points (see example in Fig 4). We then exclude voltage ranges containing these points in any model of interest (“*Disallowed Regions*” in Fig 4) from consideration and focus the fitting of *R*_*m*_ on the voltage ranges with no singularities (“*Allowed Regions*” in Fig 4). In the proposed approach, objective functions related to *R*_*m*_ are selected from allowed regions only, eliminating the discontinuity problem discussed in the previous subsection.

To define the allowed and disallowed regions for this paper, three human cardiac ventricular cell models (TNNP, different configurations of the IMW, and [28]) are used. The extent of the disallowed regions is chosen as 5 mV outside the “outermost” singularity (black pentagrams in Fig 4) of any of the models of interest. The singularity in repolarization phase for one of the considered ventricular models [28] (not shown in Fig 4) happens around -70 mV; as a result, the extension of *Disallowed Region* 2 is similar to what is seen in Fig 4. The determination of the disallowed regions is specific to the models and AP configurations of interest in a particular fitting problem and must thus be carried out for the defined fitting scenario of interest.

### Problem definition of the multi-objective optimization

The general formulation of the multi-optimization problem (MOP) is as follows:

$$\begin{array}{l} \underset{x_{i}}{\min}F\left(x\right)\\ s.t.\;x_{i}\in X,i=1,\ldots ,p\\ \;H(x_{i})=0\\ \;G(x_{i})\leq 0\\ \;{lb}_{i}\leq x_{i}\leq {ub}_{i} \end{array}$$ (1)

where *F*(*x*) = [*f*_*1*_(*x*),*…*, *f*_*M*_]^*T*^ is a vector of objective functions, and *M* is the number of objectives thereof, that varies by the user preference. *x*_*i*_ (*i* = 1,…, *p*) is a vector of decision variables, *G*(*x*_*i*_) and *H*(*x*_*i*_) are inequality and equality constraint vectors, respectively. *ub*_*i*_ and *lb*_*i*_ are considered as vectors of upper and lower bounds of decision variables, respectively. In the following paragraphs, firstly, the objective functions defined in this study are introduced. Thereafter, decision variables utilized in our MOP are demonstrated.

*F*(*x*) reflects cellular and intercellular parameters. Amid cellular properties, minimizing the root-mean-squared-error (RMSE) of AP is considered as the first objective. The RMSE of AP (*RMSE*_*AP*_) can be calculated as follows:

$$f_{1}\left(x\right)=\sqrt{\frac{1}{G}{\sum_{i=1}^{G}(V^{reference}\;\left(iT_{s}\right)-V^{fitting\;result}\;\left(iT_{s}\right))}^{2}}$$ (2)

where *G* and *T*_*s*_ denote the number of sample points and sample time, respectively. *V* ^*reference*^ and *V* ^*fitting result*^ are the AP waveforms of the *reference model* and fitted *base model*, respectively.

Fitting selected points of the *R*_*m*_ profile in the allowed regions, discussed in the previous subsection, could be taken into consideration as a key intercellular property. In this study, errors in fitting several points within *Allowed Region* 2 are considered as potential objectives of the optimization problem. However, considering multiple resistance points increases the dimension of the optimization problem, thereby increasing the computational complexity. Hence, we instead settle on the curve of *R*_*m*_ profile obtained in *Allowed Region* 2 as the second objective. To calculate this curve, first, the voltage clamp protocol described in *R*_*m*_ measurement subsection is applied to the AP waveform within *Allowed Region* 2 for each 5 mV. Then, the calculated discrete points of *R*_*m*_ are connected by interpolation to produce a more densely sampled *R*_*c*_ curve. The RMSE of *R*_*c*_ (*RMSE*_*Rc*_) as a second objective is then obtained as follows:

$$f_{2}\left(x\right)=\sqrt{\frac{1}{D}{\sum_{j=1}^{D}\left(R_{c}^{reference}\;\left(j\right)-R_{c}^{fitting\;result}\;\left(j\right)\right)}^{2}}$$ (3)

where *D* is the number of discrete points obtained by interpolation. *R*_*c*_^*reference*^ and *R*_*c*_^*fitting result*^ are the reconstructed *R*_*m*_ points in *Allowed region* 2, for the *reference model* and fitted *base model*, respectively.

The value of *R*_*m*_ in the diastolic (resting) phase (*R*_*d*_) in *Allowed Region* 3 is another intercellular property derived from the *R*_*m*_ profile. The absolute error (AE) of *R*_*d*_ (*AE*_*Rd*_) is utilized as a third objective:

$$f_{3}\left(x\right)=abs\left(R_{d}^{reference}-R_{d}^{fitting\;result}\right)$$ (4)

where *R*_*d*_^*reference*^ and *R*_*d*_^*fitting result*^ are the *R*_*m*_ values in *Allowed Region* 3 of the *reference model* and fitted *base model*, respectively.

Based on the above description, three objective functions are introduced, in which *M* varies regarding the scenarios defined in the description of optimization scenarios subsection.

The *x*_*i*_ (*i* = 1,…,16) are *G*_*Na*_, *G*_*CaL*_, *G*_*bNa*_, *G*_*bCa*_, *G*_*Kr*_, *G*_*to*_, *G*_*K1*_, *G*_*Ks*_, *G*_*pk*_, *P*_*NaK*_, *k*_*NaCa*_, *G*_*pCa*_, *V*_*maxup*_, *c*_*rel*,_ *a*_*rel*_, and *V*_*leak*_ for the results presented in this paper. These parameters are maximum conductances and maximum rates for transporters, pumps, and intercellular Ca^2+^ handling mechanisms in the model. The *ub*_*i*_ and *lb*_*i*_ are defined within the physiological ranges and obtained by trial and error search [6]. More explanations of parameters and boundaries are provided in Table A1 in the S1 Appendix. Our formulation does not require equality and inequality constraints described in Eq 1.

### The solution to the multi-objective problem

**Fig 5:**
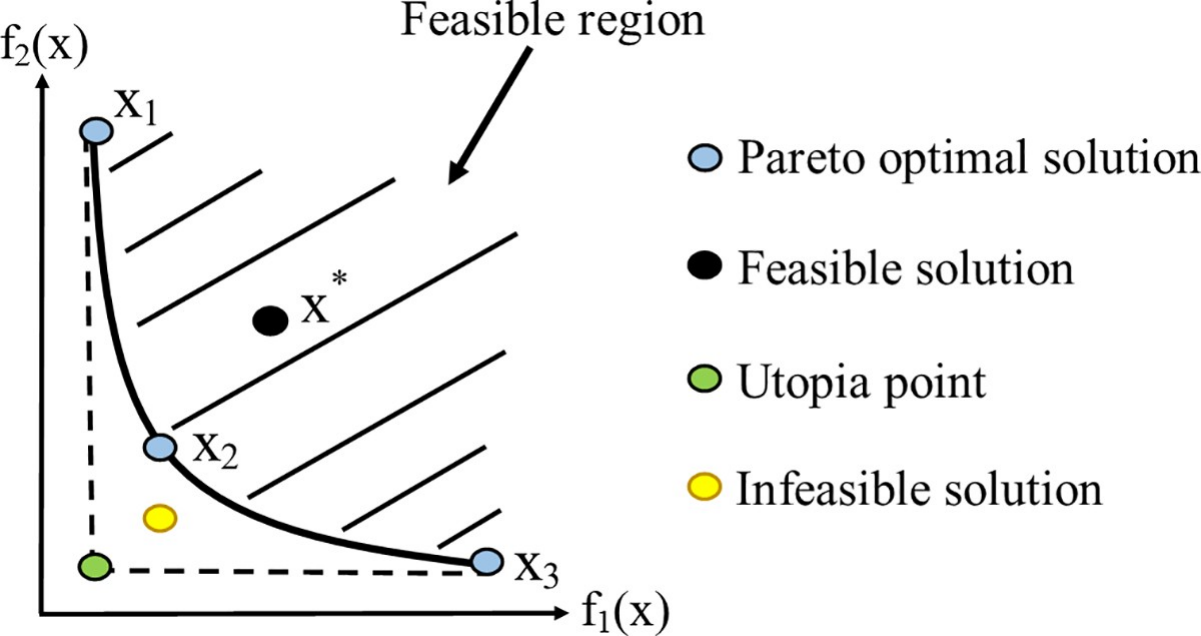
The basic concept of the MOP solution on a convex Pareto front.

The outcome of the optimization is a set of optimal solutions, in which there is a trade-off among different conflicting objectives. In other words, there are contradictions among objectives, and some of them can only be optimized while others are not at their optimum value. The basic terminologies regarding MOP solutions are represented as follows and are further explored in Fig 5.

*Definition 1- Dominant solution*: A solution set *x*_*2*_ dominates x^*^, if *f*_*i*_ (*x*_*2*_) *≤ f*_*i*_ (x^*^), *i =* 1, 2,…, *M* (where *M* is the number of objectives) and among the different inequalities, at least one of them is strict.

*Definition 2- Pareto front*: A solution that is not dominated by other solutions is a Pareto-optimal solution. The set of all Pareto solutions constitute the Pareto front.

In Fig 5, *x*_*1*_, *x*_*2*_, and *x*_*3*_ are Pareto optimal solutions; and therefore, they are among the solution sets which held the trade-off among objectives. As can be seen in this figure, Pareto optimal solutions from the Pareto front (black curve), which consisting of the solutions of the Pareto front set, are not dominated by other solutions. In the case that the Pareto front is obtained precisely, no feasible solution below the Pareto front can be attained. Therefore, the solution point, marked with yellow, is infeasible. The green point called utopia is the minimum solution for all the objectives; however, reaching this solution is infeasible. It is worth noting that the multi-objective optimization aims at attaining solution sets, forming the Pareto front approximation with minimum deviation from the true Pareto front.

### Multi-objective evolutionary algorithms background

The most general and simplest approach to solve the MOPs is the weighted-sum approach, in which the user defines the values of weights. The choice of weight values highly depends on the problems and the level of priority of each objective. In this method, the MOP is transferred to a single objective by defining a new objective function, which is the summation of each normalized weighted objective [29]. Being dependent on the weight vector, selected by trial and error in a time-consuming procedure, is amid the major drawbacks of this approach. This problem can be addressed by multi-objective evolutionary algorithms (MOEAs) in which the simultaneous search of algorithm results in finding the trade-off among objectives. A wide range of MOEAs has been proposed in the last decade to solve complex optimization problems heuristically [30, 31] in various fields of study, including biological engineering [32–34].

### NSGA-II

Amongst MOEAs, NSGA-II (non-dominated sorting genetic algorithm II) [35] is a powerful optimization tool. Some properties of this algorithm for utilizing in our problem are as follows:

1) Utilizing the elitism principle maintains the best solutions for the next generation, 2) crowding distance technique provides a diversity of the solutions, and 3) it has been demonstrated to be a successful algorithm in many engineering problems.

The definitions required to understand the procedure of NSGA-II are given as follows:

*Definition 3- Crossover*: The crossover is an operator to mix the genetic information of the parents and produce the Offspring. In NSGA-II, simulated binary crossover (SBX) is used, which creates pairs of offspring from parents based on the crossover index.

*Definition 4- Mutation*: The genetic diversity of the population from one generation to another is controlled by the operator known as mutation. In NSGA-II, the polynomial mutation is utilized, which controls the probability distribution of solutions using an external parameter. This parameter adjusts the diversity of solution sets.

*Definition 5- Crowding distance*: Crowding distance is determined by the cube enclosing each solution and measures the diversity of a solution from its neighbor. The solution with the largest crowding distance wins the tournament selection and is kept for the next iteration.

A brief description of NSGA-II procedure is as follows:

**Step 1-** Initialization: at the initial iteration, *t* = 0, the parent population of size *N* (*P*_*t*_) is randomly generated by assigning random values to the parameters within the upper and lower bounds as described in the problem definition of the multi-objective optimization subsection. Crossover and mutation operations are utilized to generate the offspring population (*Q*_*t*_) of the first iteration.

**Step 2-** Pareto fronts construction: to extract the Pareto fronts (*F*_*i*_, *i = 1*, *…*), firstly, the combined population (*R*_*t*_) consisting of *P*_*t*_ and *Q*_*t*_ is generated at each iteration. Then, non-dominated sorting is applied to each solution of the population to categorize the *R*_*t*_ into fronts. Individuals who are not dominated by others are chosen for the 1^st^ front. Such individuals that are dominated by individuals in the 1^st^ front, while they are not dominated by any other individuals, are classified as front 2. This procedure is repeated recursively to form all possible Pareto fronts.

**Step 3-** Elitist set construction: the individuals participate in the competition to be selected. The criteria to choose the elitist sets of parameters (problem definition of the multi-objective optimization subsection) are based on the rank of domination and crowding distance. For individuals in different fronts, those in the lower front win the tournament (non-dominated rank). If individuals have the same rank (same front), the individuals with larger crowding distance are selected. The elitist set is used as the parent population of the *t+1*^*th*^ iteration (*P*_*t+1*_).

**Step 4-** Satisfying the stopping criterion: mutation, crossover, and steps 2–3 are repeated until the stopping criterion is met. Here, a maximum number of iterations is set as the stopping criterion.

Finally, the 1^st^ Pareto front of *R*_*t*_ obtained from the last iteration is attained as the solution sets.

As population size and the number of evaluations greatly impact the execution time of the optimization, by performing a sensitivity analysis using a grid search and comparing the Pareto sets of various runs, 100, 10000, 20, and 20 are opted for population size, the number of evaluations, the distribution index of simulated binary crossover, and distribution index of polynomial mutation, respectively. Therefore, the results of runs pertain to this set of hyperparameters are reported in this paper.

### Selection of optimal solution

Once the Pareto front for a multi-objective optimization problem has been found, the selection of a single optimal solution as a preferred solution is essential in the decision-making process. In this work, the following equation is defined to choose a desirable solution:

$$\underset{j}{\min}\frac{{\left(\sum_{i=1}^{M}\left(\frac{f_{\min}^{i}-f_{j}^{i}}{f_{\min}^{i}-f_{\max}^{i}}\right)\right)}_{j}}{\sum_{j=1}^{N}{\left(\sum_{i=1}^{M}\left(\frac{f_{\min}^{i}-f_{j}^{i}}{f_{\min}^{i}-f_{\max}^{i}}\right)\right)}_{j}}$$ (5)

where *N* and *M* are the population size and number of objectives, respectively. *f*_*j*_^*i*^ is *i*^*th*^ objective for the *j*^*th*^ solution sets. *f* ^*i*^_*min*_, and *f* ^*i*^_*max*_ represent the minimum and maximum values of *i*^*th*^ objective, respectively [36].

## Results

### Description of considered AP configurations

**Fig 6:**
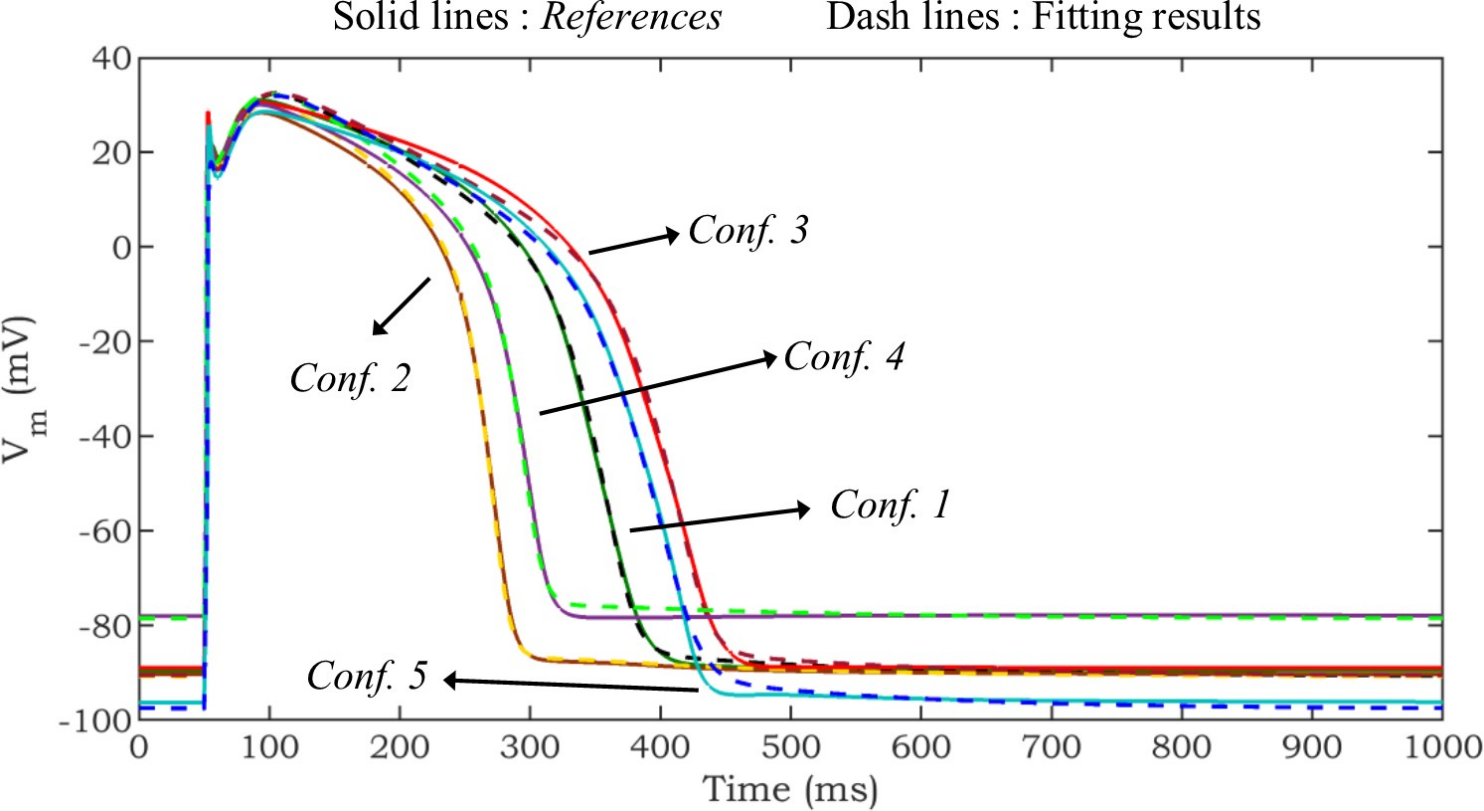
Representation of *Configurations* 1–5 and fitting results. In the optimization process, only AP is considered as an objective. Solid and dash lines show the *reference*s and fitting results, respectively.

In this paper, three scenarios consisting of different combinations of objectives (see next subsection) are utilized to examine the efficacy of the proposed optimization problem in fitting the characteristics of the *base model* to the *reference* one. In each scenario, characteristics of five distinct configurations of the IMW model (Fig 6) are considered as *reference*s to verify the robustness of the approach. In *Configuration* 1, the baseline IMW model is used as a *reference*. *Configurations* 2 and 3 explore parameter sets that produce shortened and prolonged action potential duration (APD) of the IMW model, respectively. These configurations are defined based on the crucial role of potassium (K^+^) channels in arrhythmia disease. Increased K^+^ current amplitude results in APD shortening and can induce re-entry, while decreased K^+^ current amplitude prolongs APD. EAD and a form of abnormal heart rhythm called torsades de pointes can occur due to excessive prolongation of the APD [37]. To simulate these conditions, the availability of delayed rectifier K^+^ currents (I_kr_ and I_ks_) are changed by manipulating the maximum conductances associated with these currents. *Configurations* 4 and 5 are devised to represent conditions of hyperkalemia and hypokalemia, respectively. In hyperkalemia [K^+^]_o_ is increased to above 6 mM, and in hypokalemia [K^+^]_o_ is decreased to below 3.5 mM [24]. Thus, in *Configurations* 4 and 5, [K^+^]_o_ are set to 6.5 mM and 3 mM, respectively.

As described in the problem definition of the multi-objective optimization, the RMSE and AE, well-defined evaluation criteria in the literature (e.g., in [5]), are used to assess the accuracy of the fitting.

### Description of optimization scenarios

Three distinct optimization scenarios are considered in this paper to assess the contributions of different fitting objective functions to the outcome of the optimization.

*Scenario* 1 involves single-objective optimization considering only AP fitness. The optimization is defined and solved using GA. In this scenario, *RMSE*_*AP*_ obtained by Eq 2 is the only objective function.

*Scenario* 2 is a bi-objective optimization problem with two objective functions, namely *RMSE*_*AP*_ (Eq 2) and *RMSE*_*Rc*_ (Eq 3). The optimization is solved by NSGA-II.

In *Scenario* 3, minimizing the *AE*_*Rd*_ (Eq 4) is considered as an additional objective in addition to reducing the *RMSE*_*AP*_ and *RMSE*_*Rc*_. The multi-objective optimization is solved by NSGA-II.

The relative percentage of improvement or deterioration in fit for the different scenarios is calculated as follows:

$$C=\frac{\left|E_{1}-E_{2}\right|}{E_{1}}\times 100$$ (6)

where *E*_*1*_ and *E*_*2*_ are evaluation metrics (e.g., *RMSE*_*AP*_) for the benchmark model and the model of interest for two scenarios.

For the purpose of comparing the accuracy of various scenarios in fitting *RMSE*_*Rc*_ and *AE*_*Rd*_, a posteriori calculations of *RMSE*_*Rc*_ and *AE*_*Rd*_ are conducted for *Scenario* 1, while a posteriori calculation of *AE*_*Rd*_ is performed for *Scenario* 2.

### Numerical results

**Fig 7:**
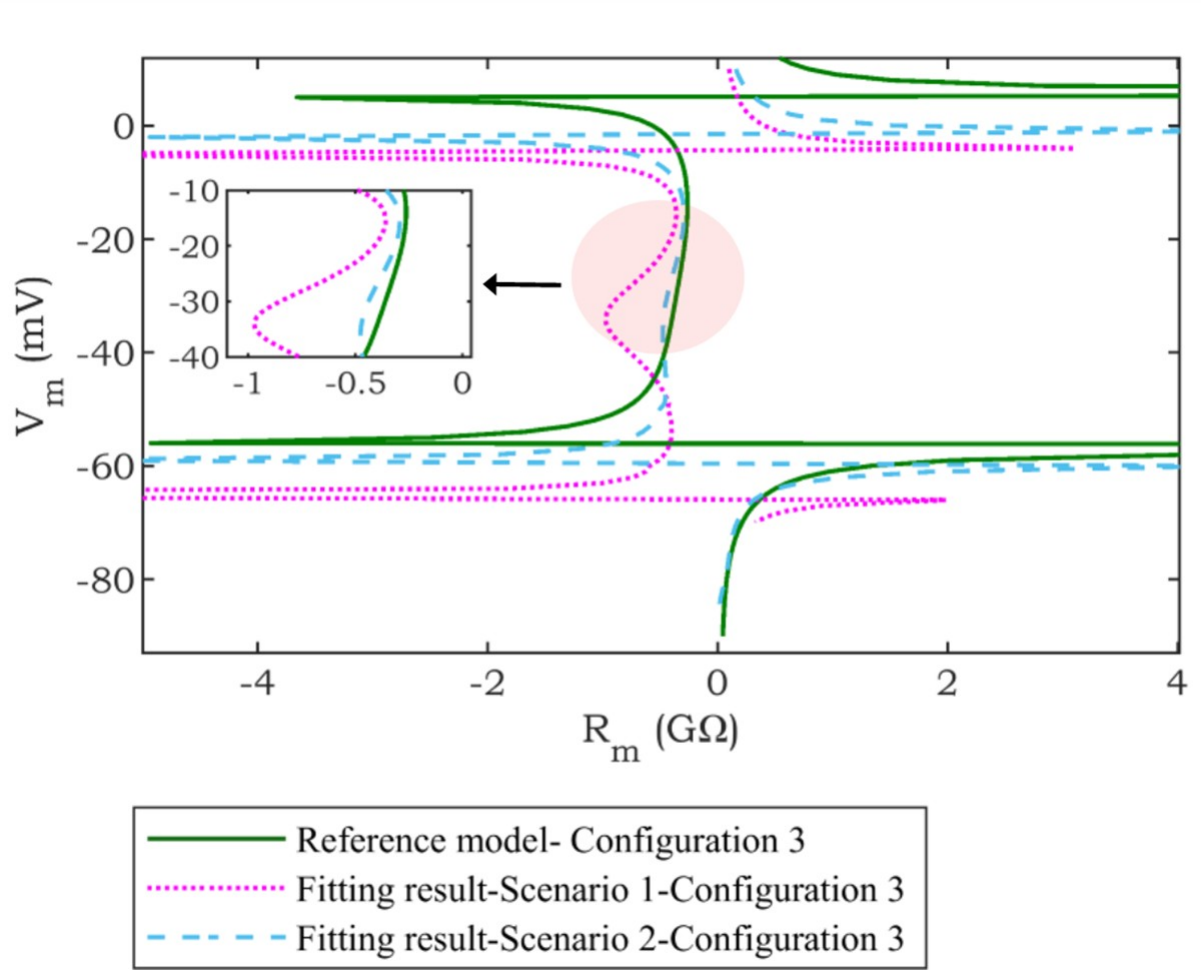
An example of a comparison between *Scenarios* 1 and 2. The results of fitting *R*_*c*_ curve (zoomed area) and *reference* model (*Configuration* 3) in *Scenarios* 1 and 2 are compared.

The average (Ave.) and standard deviation (Std.) of *RMSE*_*AP*_ (mV), *RMSE*_*Rc*_ (GΩ), and *AE*_*Rd*_ (GΩ) in 10 trials are calculated for all scenarios and configurations, and the results are presented in Table 1. In *Scenario* 1, the range of Ave. and Std. of *RMSE*_*AP*_ are changed within [1.05, 1.88] mV and [0.15, 0.28] mV, respectively. Although, the AP fit in this scenario is relatively good (Fig 6), *RMSE*_*Rc*_ is quite high. As an example, this poor-fitting *R*_*c*_ in *Scenario* 1 is demonstrated for one of the configurations in Fig 7. This figure illustrates the difference between *R*_*c*_ of the optimization result and the *reference* for one of the trials of *Scenario* 1, *Configuration* 3 (*RMSE*_*AP*_ = 1.53 mV, *RMSE*_*Rc*_ = 0.35 GΩ). This observation demonstrates that considering the AP only as an objective may not necessarily result in a good fit for intercellular properties (i.e., *R*_*c*_) to those of the *reference* (model or experimental data). As can be clearly seen from Table 1, in *Scenario* 2, the Ave. of *RMSE*_*Rc*_ in *Configurations* 1–5 are considerably improved by 89.89%, 95.05%, 88.34%, 95.52%, and 96.14%, respectively in comparison to corresponding configurations in *Scenario* 1. Fig 7 confirms this significant improvement in *RMSE*_*Rc*_ compared to *Scenario* 1. Moreover, Std. of *RMSE*_*Rc*_ in *Scenario* 1 are notably larger than the corresponding values in *Scenario* 2 (Table 1). This observation shows that fitting AP only (*Scenario* 1) does not guarantee the consistent fitting of *R*_*m*_, and generally results in high variability in *R*_*m*_ fitting. *Scenario* 2 results in a robust fitting of *R*_*m*_, as evidenced by a significant improvement in the Ave. and Std. of *RMSE*_*Rc*_ compared to *Scenario* 1. However, this comes at the expense of relatively worse results for the AP fit. Specifically, the Ave of *RMSE*_*AP*_ increases by 25.93%, 33.07%, 22.76%, 20.49%, and 30.40% in *Configurations* 1–5, respectively, compared to the corresponding values for *Scenario* 1.

**Table 1:** Performance evaluation of different scenarios for *Configurations* 1–5.

Scenarios-Configurations	*RMSE*_*AP*_ (mV)		*RMSE*_*Rc*_ (GΩ)		*AE*_*Rd*_ (GΩ)	
	Ave.	Std.	Ave.	Std.	Ave.	Std.
*Scenario* 1*-Configuration* 1	1.2561	0.1641	0.5619	0.3457	0.0335	0.0105
*Scenario* 1*-Configuration* 2	1.0508	0.1933	0.1092	0.0429	0.0224	0.0095
*Scenario* 1*-Configuration* 3	1.3843	0.1510	0.4134	0.2032	0.0350	0.0060
*Scenario* 1*-Configuration* 4	1.4397	0.2846	0.2498	0.1227	0.0266	0.0106
*Scenario* 1*-Configuration* 5	1.8784	0.2261	0.6654	0.3160	0.0917	0.0943
*Scenario* 2*-Configuration* 1	1.5818	0.2793	0.0568	0.0562	0.0363	0.0016
*Scenario* 2-*Configuration* 2	1.3983	0.0590	0.0054	0.0013	0.0368	8.48e-04
*Scenario* 2*-Configuration* 3	1.6993	0.2280	0.0482	0.0319	0.0359	0.0021
*Scenario* 2*-Configuration* 4	1.7347	0.2274	0.0112	0.0026	0.0221	0.0078
*Scenario* 2*-Configuration* 5	2.4494	0.1697	0.0257	0.0173	0.0249	0.0205
*Scenario* 3*-Configuration* 1	2.5363	0.4904	0.0954	0.1059	0.0020	0.0014
*Scenario* 3-*Configuration* 2	2.3708	0.3673	0.0160	0.0111	0.0011	7.15e-04
*Scenario* 3*-Configuration* 3	2.5978	0.4780	0.0928	0.0906	0.0015	0.0011
*Scenario* 3-*Configuration* 4	2.4428	0.2372	0.0216	0.0069	0.0019	0.0023
*Scenario* 3*-Configuration* 5	2.7903	0.4101	0.0549	0.0662	0.0013	0.0013

As shown in Table 1, in *Scenario* 3, minimization of the third objective (*AE*_*Rd*_) is realized at the expense of increasing Ave. of *RMSE*_*AP*_ by 60.34%, 69.55%, 52.87%, 40.82%, and 13.92% in *Configurations* 1–5, respectively, compared to *Scenario* 2. In this scenario, the Ave. of *RMSE*_*Rc*_ is better than in *Scenario* 1, and the Std. of *RMSE*_*Rc*_ is slightly worse than in *Scenario* 2. Importantly, depending on the specific configuration, *Scenario* 3 can result in up to 98.58% improvement (*Configuration* 5) in Ave. of *AE*_*Rd*_ compared to two other scenarios. Compared to *Scenario* 1 and 2, *Scenario* 3 yields slightly lower Std. of *AE*_*Rd*_ for all configurations.

Three trials of *Scenario* 1 for *Configuration* 1 regarding AP with similar RMSE are shown in Table 2 to compare their *RMSE*_*Rc*_. As can be discerned from Table 2, the fitting results in terms of *RMSE*_*AP*_ are comparable for all three trials; however, there is significant variability in *RMSE*_*Rc*_. Hence, this table further demonstrates that different sets of parameters can yield similar cellular property (i.e., AP), while their *R*_*m*_ values—indicators of intercellular property—are quite different.

**Table 2:** Comparison of three trials with similar *RMSE*_*AP*_ and various *RMSE*_*Rc*_ in *Scenario* 1 and *Configuration* 1.

# Trials	*RMSE*_*AP*_ (mV)	*RMSE*_*Rc*_ (GΩ)
1	1.2952	1.1102
2	1.2830	0.7590
3	1.2801	2.1144

**Fig 8:**
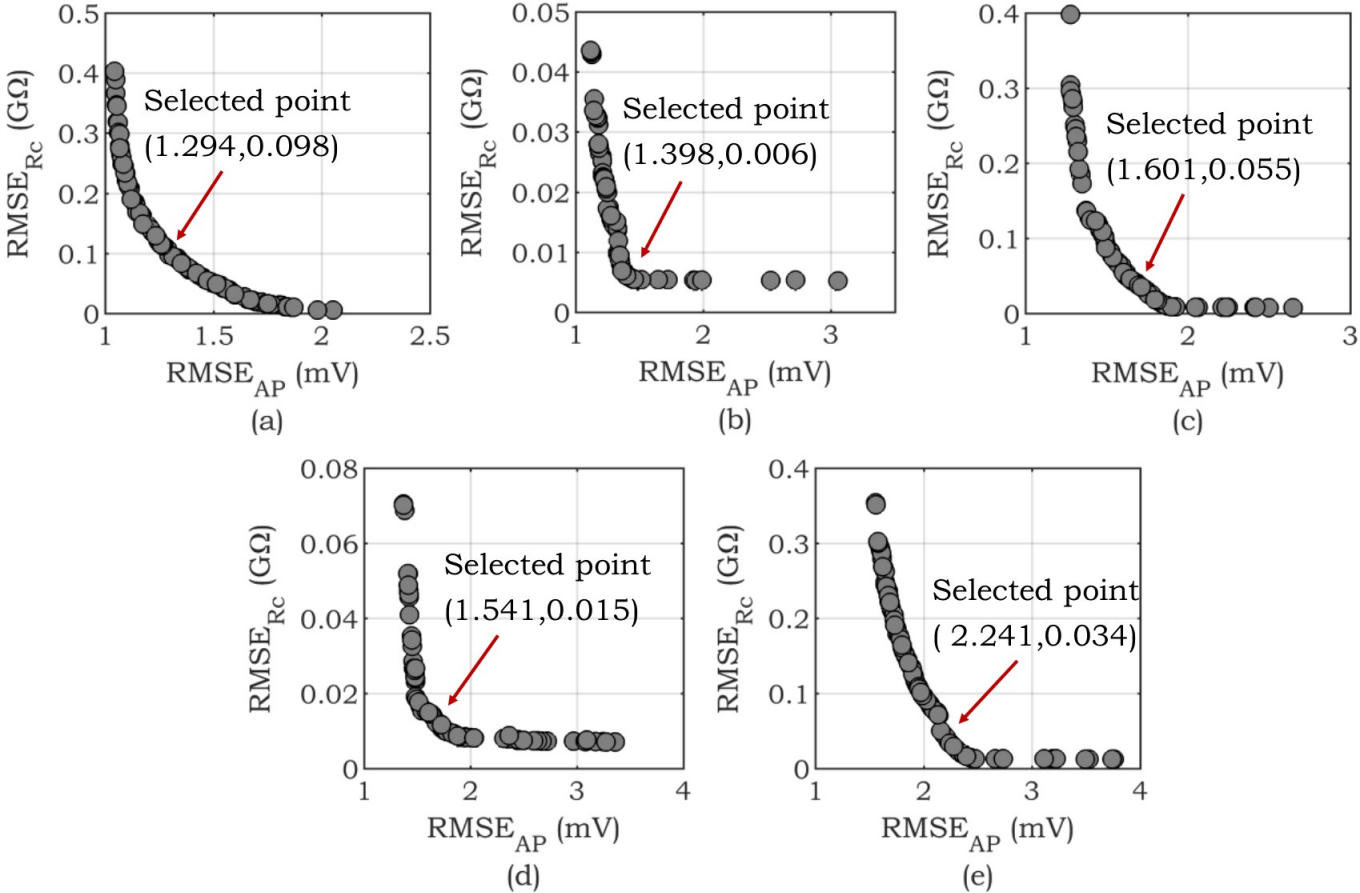
Examples of convex Pareto front in *Scenario* 2. Different configurations of the IMW model are considered as the target of optimization. a) *Configuration* 1 (Baseline), b) *Configuration* 2 (Shortened APD), c) *Configuration* 3 (Prolonged APD), d) *Configuration* 4 (Hyperkalemia condition), e) *Configuration* 5 (Hypokalemia condition). In each configuration, the desired solution is demonstrated by the red arrow.

To further examine the performance of the proposed approach, the Pareto fronts of all configurations for *Scenario* 2 are shown in Fig 8. Fig 8A–8E represents examples of the convex Pareto fronts attained from solving *Scenario* 2 for different configurations. It is clear from these figures that there is a trade-off between minimizing the *RMSE*_*AP*_ and *RMSE*_*Rc*_. The selection of the optimal solution from the Pareto front is highly dependent on the application and whether AP or *R*_*c*_ improvement is considered more important. In this general study, relying on existing literature on the applications of multi-objective optimization, we select one preferable solution as described in the selection of optimal solution subsection. It can be seen from Fig 8 that, in all configurations, the selected points yield a lower error in comparison to Ave. of *RMSE*_*Rc*_ in *Scenario* 1. Hence, these results also demonstrate that *RMSE*_*Rc*_ can be improved only by sacrificing AP fitness.

**Fig 9:**
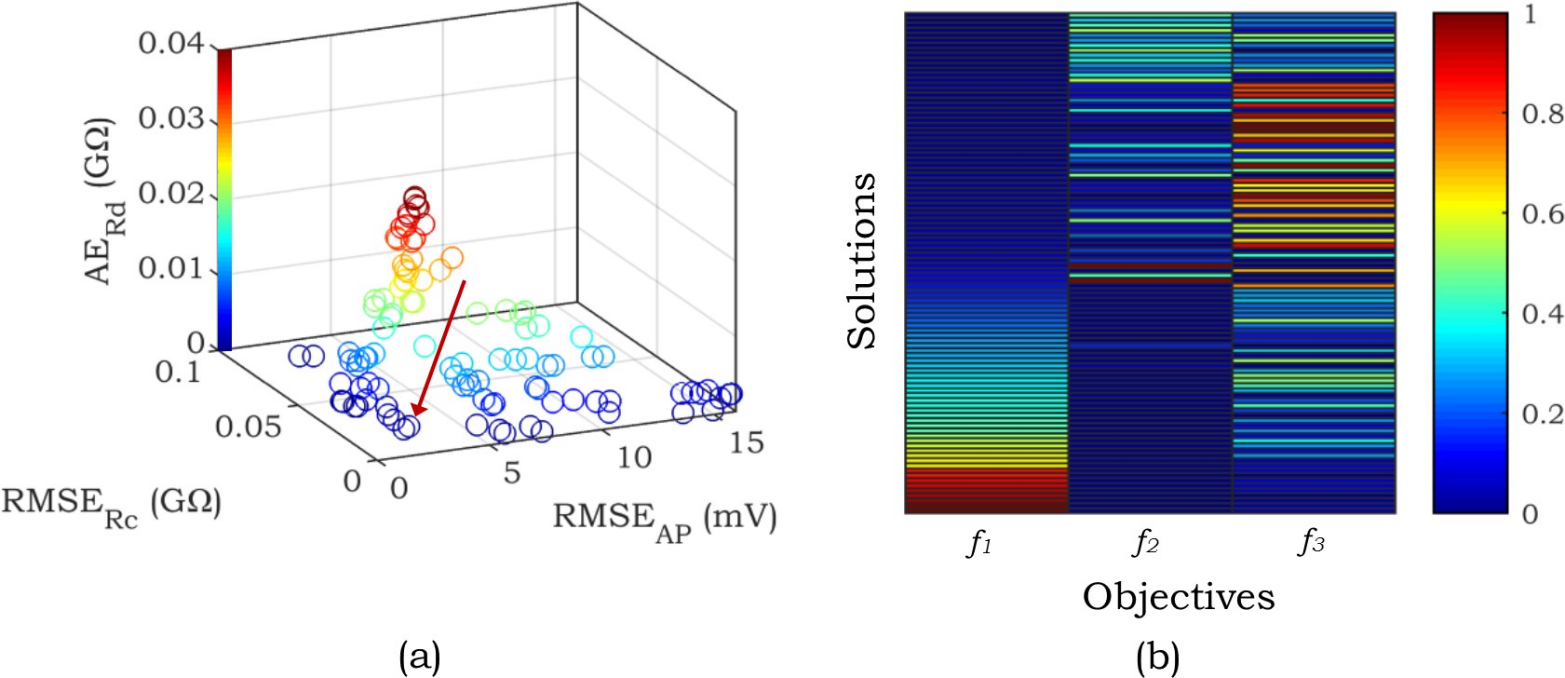
Demonstration of Pareto front in *Scenario* 3, *Configuration* 2 (Shortened APD). (a) 3D representation: The arrow demonstrates the preferred solution. The color bar shows the range of change of *AE*_*Rd*_. (b) Heatmap: The solutions are ordered according to the low to the high value of *f*_*1*_ (*RMSE*_*AP*_).

Fig 9 shows one trial of *Configuration* 2 in *Scenario* 3, illustrating the trade-off among objectives. The Pareto front obtained for this configuration is shown in Fig 9(A). In this figure, the selected solution set is indicated by an arrow (*RMSE*_*AP*_ = 2.45 mV, *RMSE*_*Rc*_ = 0.018 GΩ, and *AE*_*Rd*_ = 0.0005 GΩ) and the color scale is associated with the third objective (*AE*_*Rd*_). To further elaborate on trade-offs among objectives, the heatmap of Pareto front is demonstrated in Fig 9(B). Solutions are ordered based on the low value to the high value of the first objective (*RMSE*_*AP*_). The color of each cell shows the normalized error for the corresponding objective. The confliction of objectives can be vividly interpreted in this figure.

## Discussion

### Comparison with previous work

In this paper, a novel problem formulation for fitting human ventricular cellular models is proposed. The work presented in [3] was an important starting point and inspiration for our work. In [3], 9 parameters were optimized, and a small number of arbitrarily selected *R*_*m*_ points considered in a model-to-model fit. To obtain more accuracy, we have explored a wider range of search space by considering 16 parameters, defined in the problem definition of the multi-objective optimization, for fitting similar models to those used in [3]. The study in [3] found that AP only fitting did not produce accurate AP fits, and that also considering *R*_*m*_ resulted in improvement of the AP fit. However, with more investigation in this study, it is observed that acceptable AP fitting can be achieved using the error in the AP waveform as the only objective function with higher search space dimensions (Fig 6). Here, the proposed approach is also evaluated using four other configurations of the IMW model, including shortened APD, prolonged APD, hyperkalemia, and hypokalemia conditions.

It is important to note that in *Scenario* 2, an arbitrary selection of *R*_*m*_ values as fitness functions may be susceptible to choosing the discontinuous *R*_*m*_ points. As an improvement to the cellular model tuning proposed in [3], we define allowed regions within which *R*_*m*_ values can be used as objective functions and avoid regions near the singularities of *R*_*m*_. Thus, the *R*_*m*_ points are selected from regions that are less vulnerable to producing errors in model fitting. Moreover, in this work, the number of objectives is reduced by curve fitting instead of fitting the distinct points of *R*_*m*_. This reduction simplifies the optimization and yields enhanced decision-making ability on the final solution.

### Trade-offs among objectives

In this work, we have fitted up to three different objective functions, *RMSE*_*AP*_ (mV), *RMSE*_*Rc*_ (GΩ), and *AE*_*Rd*_ (GΩ). The RMS error in matching the resultant curve is considered as a second fitting objective function. The main reason to select the *R*_*m*_ curve in *Allowed Region* 2 (*RMSE*_*Rc*_) as one of the objective functions is that negative values of *R*_*m*_ and ANOR phenomenon emerge in this voltage range [17–19]. The convex Pareto-front in the bi-objective optimization problem solved by NSGA-II is demonstrated (Fig 8) to select a preferred solution based on the application. Constraining the fitting problem by increasing the number of objective functions (i.e., *RMSE*_*Rc*_) results in a substantial reduction of average Std. of *RMSE*_*Rc*_ fitting (Table 1). This reduction demonstrates the repeatability of the algorithm across trials. The importance of *R*_*d*_ in determining the conduction velocity *θ*–one of the intercellular properties–and its relationship with [K^+^]_o_ is discussed in [23, 24]. This is a justification for the importance of *R*_*d*_ and consideration of *AE*_*Rd*_ as an objective in the proposed problem formulation. Our observations reveal that there tends to be a trade-off between this property and the two other features considered, i.e., *RMSE*_*AP*_ and *RMSE*_*Rc*_. Fig 9 shows that improvements in terms of minimizing *R*_*d*_ are feasible by sacrificing the accuracy in fitting AP and/or *R*_*c*_. However, the Ave. and Std. of *RMSE*_*Rc*_ in this scenario is still improved in comparison with *Scenario* 1.

Further investigation is required to clarify the best decision-making scheme for selecting the final solution from the Pareto front. The careful selection of additional voltage-dependent parameters and their consideration in the optimization may result in further improvement in the fitting results as it may provide an opportunity to better align the voltage-dependence of the *R*_*m*_ profile with the *reference* data. However, additional experiments that are beyond the scope of this study would be required to determine whether this would indeed improve overall fitting results.

### Future work

A key limitation of the current work is the requirement for the selection of allowed and disallowed regions, which depend on the specific models and configurations used. As a future development, it would be desirable to develop a generalized algorithm to eliminate the dependency on the designers’ somewhat subjective decisions in this regard.

The model fitting can involve some degree of uncertainty due to the variability of observations. Populations of models (POMs) are used to model the inherent variability of experimental data [38]. As a further study, the proposed framework for *R*_*m*_ could be extended to POM calibration. Considering *R*_*m*_ features might reduce the degree of uncertainty in POM as it can provide intercellular information.

## Conclusion

In this study, the benefits of including *R*_*m*_ values in cardiac cellular fitting are clearly demonstrated. We have also identified important technical challenges related to the occurrence of discontinuities in *R*_*m*_ and the voltage dependence of the *R*_*m*_ profile. These need to be addressed in the formulation of the optimization problem, or significant errors may result. This work presents and evaluates a new problem formulation for tuning specified parameters of cell models. Importantly, we provide information about the trade-offs among fitting objectives and demonstrate that it is possible to achieve a good balance among key cellular and intercellular properties. In conclusion, this paper contributes toward making mathematical models of human ventricular cells (and cardiac cells in general) more reliable by developing a robust optimization formulation for cardiac cellular fitting.

## Supporting information

S1 Appendix Overview of cardiac cellular model parameters. (DOCX)

S1 Text Description of data. (DOCX)

S1 File Data. (ZIP)
