# Supplementary material for: A robust multi-objective optimization framework to capture both cellular and intercellular properties in cardiac cellular model tuning: Analyzing different regions of membrane resistance profile in parameter fitting
Source: PLoS One. 2019 Nov 15;14(11):e0225245. doi: 10.1371/journal.pone.0225245 (PMC6857942; doi:10.1371/journal.pone.0225245)
Supplement: S1 Appendix — (DOCX) [file pone.0225245.s001.docx]

# Appendix

## Overview of cardiac cellular model parameters

In the *Base model*, AP waveform (*V_m_*) is related to ionic transmembrane currents (*I_i_*) by the following ODE:

| $\frac{dv}{dt}=\frac{-1}{c_{m}}\sum_{i=1}^{q} I_{i}(t)$ | (A.1) |
| --- | --- |

where *C_m_* is membrane capacitance, *Ii* (*i*=1,…,*q*) is the summation of different currents, including, *I_Na_*, *I_bNa_*, *I_to_*, *I_Kr_*, *I_Ks_*, *I_K1_*, *I_CaL_*, *I_bCa_*, *I_NaK_*, *I_NaCa_*, *I_pK_*, and *I_pCa_*_._ It is revealed, the parameters demonstrated in Table A1 have significant roles in the model’s behaviors [1]. In this regard, these parameters are selected as the decision variables for the multi-objective problem. The definitions [2], and boundaries [2, 3, 4] of each decision variable are illustrated in Table A1.

**Table A1. Representation of decision variables, boundaries, and definitions.**

| Parameters | Ranges | Definitions | Units |
| --- | --- | --- | --- |
| *G_Na_* | [3.8 15] | Maximal *I_Na_* conductance | *nS/pF* |
| *G_bNa_* | [1e-8 3.1-4] | Maximal *I_bNa_* conductance | *nS/pF* |
| *G_to_* | [0.09 0.4] | Maximal *I_to_* conductance | *nS/pF* |
| *G_Kr_* | [0.009 0.42] | Maximal *I_Kr_* conductance | *nS/pF* |
| *G_Ks_* | [1e-5 0.5] | Maximal *I_Ks_* conductance | *nS/pF* |
| *G_K1_* | [0.1 6] | Maximal *I_K1_* conductance | *nS/pF* |
| *G_CaL_* | [9e-5 3e-4] | Maximal *I_CaL_* conductance | *cm^3^/*$\mu$*F.s* |
| *G_bCa_* | [1e-7 1e-3] | Maximal *I_bCa_* conductance | *nS/pF* |
| *P_NaK_* | [1 30] | Maximal *I_NaK_* | *pA/pF* |
| *K_NaCa_* | [950 1100] | Maximal *I_NaCa_* | *pA/pF* |
| *V_maxup_* | [1e-4 9e-4] | Maximal *I_up_* | *mM/ms* |
| *G_pCa_* | [0.1 0.9] | Maximum *I_pCa_* conductance | *pA/pF* |
| *G_pk_* | [0.009 0.02] | Maximum *I_pK_* current | *nS/pF* |
| *a_rel_* | [0.009 0.02] | Maximal Ca-SR dependent *I_rel_* | *mM/ms* |
| *c_rel_* | [0.001 0.009] | Maximal Ca-SR independent *I_rel_* | *mM/ms* |
| *V_leak_* | [9e-6 9e-5] | Maximal *I_leak_* | *ms^-1^* |

**References**

1. Sarkar AX, Sobie EA. Regression analysis for constraining free parameters in electrophysiological models of cardiac cells. PLoS Computational Biology. 2010 Sep 2;6(9):e1000914.

2. Ten Tusscher KH, Noble D, Noble PJ, Panfilov AV. A model for human ventricular tissue. American Journal of Physiology Heart and Circulatory Physiology. 2004 Apr;286(4):H1573-89.

3. Iyer V, Mazhari R, Winslow RL. A computational model of the human left-ventricular epicardial myocyte. Biophysical Journal. 2004 Sep 1;87(3):1507-25.

4. O'Hara T, Virág L, Varró A, Rudy Y. Simulation of the undiseased human cardiac ventricular action potential: model formulation and experimental validation. PLoS Computational Biology. 2011 May 26;7(5):e1002061.
