# Supplementary material for: A robust multi-objective optimization framework to capture both cellular and intercellular properties in cardiac cellular model tuning: Analyzing different regions of membrane resistance profile in parameter fitting
Source: PLoS One. 2019 Nov 15;14(11):e0225245. doi: 10.1371/journal.pone.0225245 (PMC6857942; doi:10.1371/journal.pone.0225245)
Supplement: S1 Text — (DOCX) [file pone.0225245.s002.docx]

**DATA:**

All the data files are in MAT format and can be read with MATLAB.

-Table1_Data folder: includes 15 data structures, each structure illustrates the data behind the mean and standard deviation for all scenarios and configurations reported in Table 1 of the manuscript. The relevant descriptions are provided within the data structures.

-Examples_Configurations folder: includes 15 structures, for Scenarios 1-3 and Configurations 1-5. Please note that Scenario 1-3 are associated with the single objective, two-objective, and three-objective optimization problems, respectively. The data structures contain both objective sets with their corresponding parameter sets.

-Parameter set related to Figure 3, fitting result of AP and *R_m_* is available in the Examples_Configurations folder (S1_C1).

-Parameter sets related to Figure 6 (fitting results) are available in the Examples_Configurations folder (S1_C1, S1_C2, S1_C3, S1_C4, and S1_C5).

-Parameter sets related to Figure 7 (*R_m_* profile of Scenarios 1 and 2, Configuration 3) are available in the Examples_Configurations folder (S1_C3, and S2_C3).

-Data related to Figure 8, and 9 can be obtained in the Examples_Configurations folder (S2_C1, S2_C2, S2_C3, S2_C4, S2_C5, and S3_C3).

**Guideline for the Simulation:**

- The codes of the models are available at www.cellml.org.

-Solve the ODE for 30000 ms without stimulus, consider the resultant state variables, then produce AP by 2 ms current pulse with 20 pA/pF amplitude [1]. The model is allowed to run 20 beats, and the 20th AP is selected.

-NSGAII toolbox is provided by authors in [2].

[1] Sarkar AX, Sobie EA. Regression analysis for constraining free parameters in electrophysiological models of cardiac cells. PLoS computational biology. 2010 Sep 2;6(9):e1000914.

[2] Tian Y, Cheng R, Zhang X, Jin Y. PlatEMO: A MATLAB platform for evolutionary multi-objective optimization [educational forum]. IEEE Computational Intelligence Magazine. 2017 Oct 11;12(4):73-87.
